# Thrombospondin-4 increases with the severity of peripheral arterial disease and is associated with diabetes

**DOI:** 10.1007/s00380-019-01453-7

**Published:** 2019-06-21

**Authors:** Bernhard Zierfuss, Clemens Höbaus, Carsten T. Herz, Gerfried Pesau, Renate Koppensteiner, Gerit-Holger Schernthaner

**Affiliations:** 1 Division of Angiology, Department of Internal Medicine 2, Medical University of Vienna, Währinger Gürtel 18-20, 1090 Vienna, Austria; 2 Division of Endocrinology and Metabolism, Department of Internal Medicine 3, Medical University of Vienna, Vienna, Austria

**Keywords:** Atherosclerosis, Peripheral arterial disease, Lower extremity arterial disease, Diabetes, Biomarker, Extracellular matrix protein

## Abstract

Thrombospondin-4 (TSP-4) is an extracellular matrix protein of the vessel wall. Despite bench evidence, its significance in the clinical setting of atherosclerosis is missing. TSP-4 (ng/ml) was measured in 365 PAD patientsusing a commercially available ELISA. PAD was diagnosed by the ankle–brachial index (ABI) and clinically graded using the Fontaine classification. TSP-4 levels were significantly higher in Fontaine II vs. Fontaine I (4.78 ± 0. 42, 4.69 ± 0.42, *p* = 0.043). TSP-4 significantly correlated with ABI (*r* = − 0.141, *p* = 0.023, *n* = 259) after the exclusion of mediasclerotic patients. Binary logistic regression analysis for Fontaine I vs. II showed an OR of 1.70 (1.02–2.82) in a multivariable model adjusted for traditional risk factors. Interestingly, TSP-4 levels were higher in patients with type 2 diabetes mellitus or prediabetes (DGT) compared with normal glucose tolerance (NGT) (4.76 ± 0.42 vs. 4.66 ± 0.41, *p* = 0.035). ANOVA for PAD and diabetes subgroups showed a linear increase with disease burden with the highest difference between Fontaine I-NGT and Fontaine II-DGT (4.59 ± 0.40, 4.79 ± 0.43, *p* = 0.015). TSP-4 levels increased with PAD severity and showed a former unknown association with diabetes. Thus, TSP-4 could be a novel marker of atherosclerotic activity, especially in the major subgroup of patients with concomitant diabetes.

## Introduction

Peripheral arterial disease (PAD) is caused by atherosclerosis of the lower limb arteries in over 90% of patients. PAD itself can be considered a marker for systemic atherosclerotic processes [1]. On the pathophysiological level, atherosclerosis is postulated to be an inflammatory process promoted by several given and some modifiable risk factors such as hypercholesterinaemia, diabetes, smoking and arterial hypertension [2]. Interestingly, not every patient with impaired lower extremity perfusion (defined as an ABI < 0.9) develops symptoms of intermittent claudication (IC) [3]. Specific determinants of the development of symptoms on the other hand are not fully understood yet.

The ability to form novel vessels (angiogenesis) is hypothesized to be a major contributor in relieving IC symptoms [4]. On the other hand, a major characteristic of atherosclerotic progression into a vulnerable plaque is intraplaque haemorrhage caused by immature intraplaque vessel formation [5]. Additionally, there is no consensus on how to measure angiogenesis in the clinical setting.

Thrombospondin-4 (TSP-4) is a member of the thrombospondin family. It was first described in humans in 1995 as a homopentamer of 140 kDa [6]. Expression of TSP-4 could be observed in neuronal tissues [7], the eye [8], connective tissue [9], cardiac cells [10] and the endothelium [11]. As a matricellular protein, by the definition of Bornstein et al. [12], TSP-4 mediates various cell–cell interactions. In the vasculature, it modulates endothelial cell and smooth muscle cell proliferation. Recently, the proangiogenic effects of TSP-4 were linked with the induction of the TGF-beta pathway [13, 14].

Several studies showed a linkage of TSP-4 to the cardiovascular system. It is upregulated during cardiac pressure overload [15] and TSP-4 deficiency resulted in increased fibrosis and remodelling of the myocardium in rats [10]. In a hypertension mouse model, TSP-4 deficiency increased cardiac hypertrophy and inflammation, leading to coronary perivascular fibrosis [16]. The same study found aortic dissections to be associated with TSP-4 deficiency in a mouse model. Deficiency of TSP-4 reduced atherosclerotic plaque formation and migration of monocytes into plaques, suggesting the involvement of TSP-4 in the promotion of the atherosclerotic inflammatory process [17].

A variety of genetic studies linked a TSP-4 gene polymorphism to myocardial infarction in humans [18–20]. While several studies on the involvement of TSP-4 in atherosclerotic processes have been performed at the cellular level and in animal models, data about humans under pathologic conditions are still missing. Thus, this study aimed to examine a potential association of circulating levels of TSP-4 with peripheral arterial disease severity as an occurrence of atherosclerosis.

## Materials and methods

### Study design and patients

This study was approved by the institutional ethics committee and follows the Declaration of Helsinki and Good Clinical Practice. Frozen blood samples from the VMC Vienna cohort [21] were used for this study. Patient recruitment and characteristics have been previously described [21]. In brief, patients with stable PAD (Fontaine I–II) ranging from 40–90 years were included in this cohort. Exclusion criteria at study entry were Fontaine III or IV, cancer or critical illness within the last 6 months, serum creatinine > 3 mg/dl, type 1 diabetes mellitus, connective tissue disease and hormone replacement therapy.

In patients without a history of vascular interventions, PAD was defined by an ankle–brachial index (ABI) below 0.9 according to TASC II criteria [22] and classified according to the Fontaine classification. ABI determination was performed by specially trained technical staff. Mönckeberg’s mediasclerosis was assessed by an ABI > 1.4 or incompressible arteries. In those patients, PAD was defined by a toe-brachial index (TBI) < 0.70, which is the generally accepted cutoff value. In 8 patients, toe pressure values were too low to measure valid oscillometric curves (assumed by a toe pressure < 30 mmHg). TBI was set to 0.1 in this case. Those patients were excluded from correlation analysis. The term PAD in this manuscript reflects the term lower extremity atrial disease (LEAD) used in the recent European Society of Cardiology PAD guidelines. Other forms of PAD including upper extremity, cerebral, or visceral artery disease were not used as inclusion criteria. According to the ADA criteria of 2009 [23], type 2 diabetes mellitus was diagnosed by a fasting glucose level ≥ 126 mg/dl (> 7 mmol/l) or a glucose level  ≥ 200 mg/dl (11.1 mmol/l) 2 h after an oral load of 75 g glucose dissolved in water (OGTT). Impaired fasting glucose (IFG) was defined by fasting glucose between 100 and 125 mg/dl. Impaired glucose tolerance (IGT) was defined by a glucose level between 140 and 199 mg/dl 2 h after OGTT intake. Patients were categorized as normal glucose tolerance (NGT) or disturbed glucose tolerance (DGT), a combination of prediabetes (IFG, IGT) and T2DM. Hypertension was defined by a systolic blood pressure above 140 mmHg and by a diastolic pressure above 90 mmHg in at least two measurements or active usage of antihypertensive medication, respectively. Urine albumin creatinine ratio (UACR) > 30 mg/g was defined as micro-albuminuria and > 300 mg/g as macro-albuminuria using spot urine. The Chronic Kidney Disease Epidemiology Collaboration (CKD-EPI) [24] formula was used for the calculation of estimated glomerular filtration rate (eGFR). Creatinine clearance was calculated by the Cockroft–Gault formula.

### Measurement of TSP-4

At study entry, serum samples were collected after an overnight fast, immediately centrifuged and stored at − 80 °C until measurement. After overnight thawing, plasma TSP-4 values (ng/ml) were measured using a precoated sandwich enzyme-linked immunoassay (ELISA) (Cusabio Life Sciences, Wuhan, China). Calculated intra-assay and inter-assay coefficient of variation for TSP-4 were 9.3% and 7.7%, respectively.

### Statistics

The entire statistical analyses were performed with SPSS 25.0 (SPSS Inc., Chicago, IL). Data are presented as mean ± standard deviation (SD) or median and percentiles (25th, 75th), as appropriate. Analyses included Student’s *t* test, Chi-square test, ANOVA, Kruskal–Wallis test, Mann–Whitney *U* test, univariate and multivariable correlation and regression analyses. A two-sided alpha level of < 0.05 was considered statistically significant. TSP-4 values revealed a right-skewed non-Gaussian distribution and were therefore logarithmically transformed for parametric testing. Logarithmic TSP-4 values showed a normal distribution in the Kolmogorov–Smirnov test (*p* = 0.20) and were further used throughout the manuscript. ANOVA used for group analyses for the combined groups of Fontaine stage and diabetes status (NGT and DGT) showed a non-significant Levene test (*p* = 0.781) indicating a homogeneity of variances and Tukey’s HSD test was therefore used for post hoc analysis. In one patient, data on glucose monitoring was missing. Thus, only 364 patients were included into this analysis. Smoking was defined as never versus former or active use in multivariable models. For better comparability and since no standard values for TSP-4 are defined, data and figures are presented with logarithmic TSP-4 values.

## Results

### Baseline characteristics

365 patients were enrolled into this study. There was no significant difference in traditional cardiovascular risk factor for patients according to TSP-4 tertiles. A trend was seen with increased age (*p* = 0.052), reduced creatinine clearance (*p* = 0.087), reduced eGFR (*p* = 0.075) and the combination of prediabetes and type 2 diabetes mellitus (DGT, *p* = 0.073) in the highest versus the lowest tertile. The results are presented in detail in Table 1.

**Table 1:** Baseline characteristics for patients divided into TSP-4 tertiles

		TSP-4 tertiles				
		1	2	3	*p* value	
		76.25 (65.22, 85.04)	115.57 (104.09, 124.87)	180.74 (150.77, 208.27)	overall	1 vs. 3
*n*		121	123	121		
Male Gender *n* (%)		82 (67.8%)	76	85 (70.2%)	0.354	0.677
Age		68 ± 11	68 ± 10	71 ± 10	0.097	0.052
LDL-C (mg/dl)		110.5 ± 39.4	110.1 ± 40.4	103.8 ± 36.1	0.326	0.377
HDL-C (mg/dl)		52.9 ± 14.1	53.6 ± 14.3	52 ± 12.8	0.662	0.628
Lipoprotein(a) (mg/dl)		22.0 (10.0, 75.0)	25.5 (7.0, 87.0)	25.0 (13.0, 78.0)	0.781	0.450
BMI (kg/m²)		27.4 ± 4.3	27.95 ± 4.37	27.1 ± 3.7	0.294	0.860
CRP (mg/dl)		0.27 (0.14, 0.55)	0.3 (0.14, 0.55)	0.28 (0.16, 0.56)	0.840	0.539
eGFR (ml/min/1.73 m^2^)		69.3 ± 20	67.5 ± 18.8	64.9 ± 17.7	0.2	0.075
Creatinine clearance (ml/min)		76 ± 29	75 ± 27	69 ± 24	0.081	0.087
UACR (mg/g)		10.7 (5.3, 37.3)	9.7 (4.7, 27.4)	10.7 (4.7, 27.6)	0.714	0.531
Triglycerides (mg/dl)		157 ± 91	174.2 ± 112.4	164.9 ± 82.7	0.398	0.820
HbA1c (%)		6.3 ± 0.9	6.3 ± 0.9	6.2 ± 1	0.997	0.998
Arterial hypertension *n* (%)		111 (91.7%)	114 (92.7%)	111 (91.7%)	0.951	1
Diabetes *n* (%)	NGT	42 (34.7%)	30 (24.4%)	29 (24.2%)	0.112	0.073
	DGT	79 (65.3%)	93 (75.6%)	91 (75.8%)		
Smoking habit *n* (%)	NEVER	21 (17.4%)	27 (22%)	24 (19.8%)	0.768	0.635
	FORMER	53 (43.8%)	57 (46.3%)	57 (47.1%)		
	ACTIVE	47 (38.8%)	39 (31.7%)	40 (33.1%)		
Mediasclerosis *n* (%)		29 (24%)	30 (24.4%)	33 (27.3%)	0.814	0.556
ABI		0.79 ± 0.17	0.73 ± 0.18	0.72 ± 0.21	**0.031**	**0.012**
TBI		0.52 ± 0.20	0.45 ± 0.14	0.47 ± 0.19	0.387	0.347
PAD stage *n* (%)	I	65 (54.2%)	63 (51.2%)	56 (46.3%)	0.5	0.247
	II	56 (46.3%)	60 (48.8%)	65 (53.7%)		

*ABI* is only presented for patients without mediasclerosis, *TBI* is only presented for patients with mediasclerosis, *NGT* normal glucose tolerance, *DGT* disturbed glucose tolerance (prediabetes and diabetes combined), *LDL-C* low-density lipoprotein cholesterol, *HDL-C* high-density lipoprotein cholesterol, *BMI* body mass index, *CRP* c-reactive protein, *eGFR* estimated glomerular filtration rate, *UACR* urine albumin creatinine ratio, *HbA1c* haemoglobin A1c, *ABI* ankle–brachial index, *TBI* toe-brachial index

*p* < 0.05 significant (in bold)

### Univariate associations

Variables available from Table 1 were included into the correlation analysis. Only fasting glucose showed a trend with TSP-4 (*r* = 0.092, *p* = 0.079). Furthermore, no association could be seen in cardiovascular risk factors such as age (*r* = 0.069, *p* = 0.187), HbA1C (*r* = 0.064, *p* = 0.362), CRP (*r* = 0.054, *p* = 0.314), LDL-C (*r* = − 0.74, *p* = 0.161), BMI (*r* = 0.026, *p *= 0.620) and hypertension (*p* = 0.835).

Since univariate associations suggest a trend for TSP-4 and diabetes, a univariate regression for this combination was performed. Diabetes status was significantly associated with TSP-4 (beta = 0.11, *p* = 0.035) with an *R*^2^ of 0.012. We failed to find a stronger explanatory model for TSP-4.

### TSP-4 and PAD

**Fig. 1:**
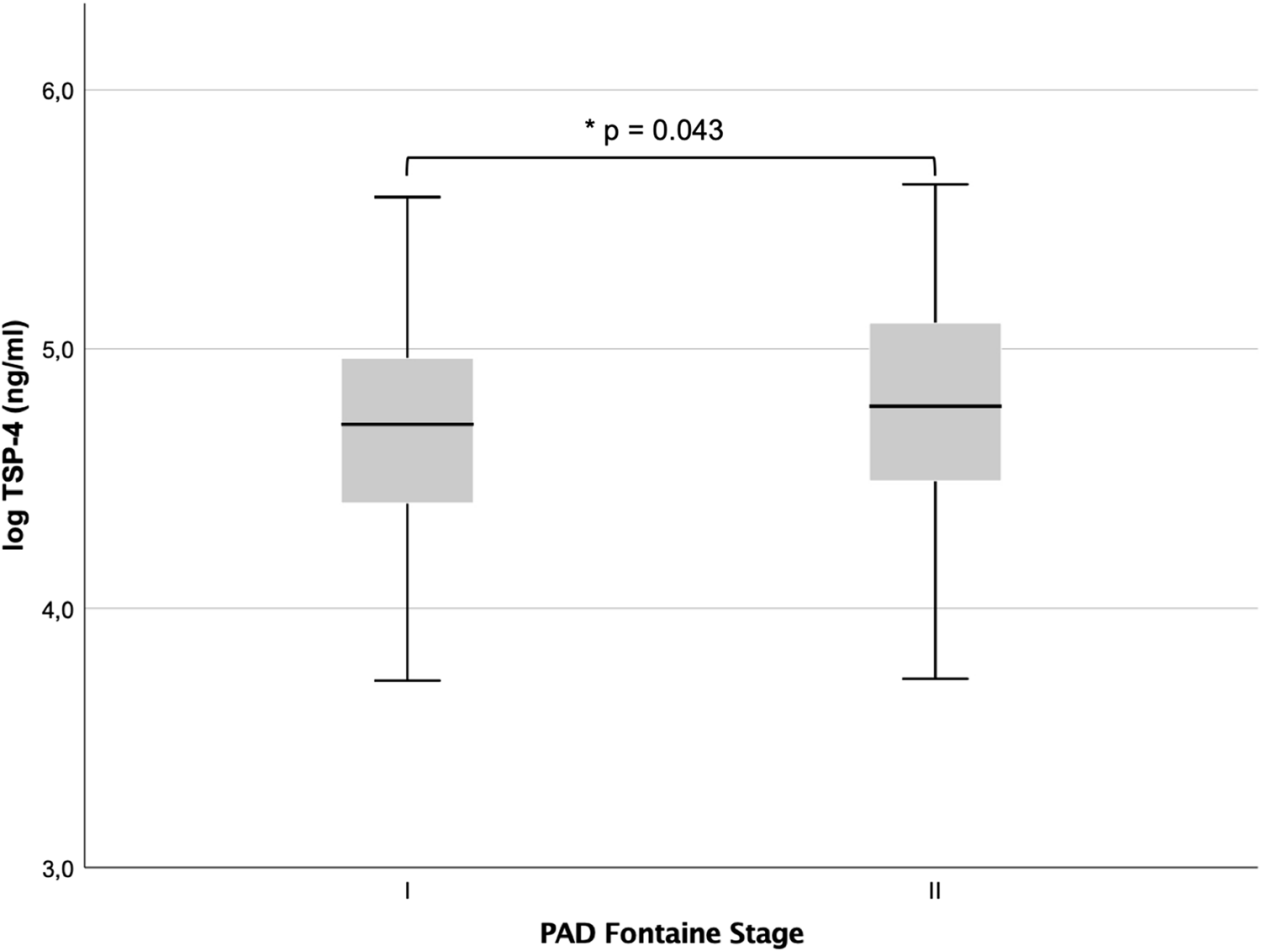
Boxplot for PAD I vs. II shows a significant difference in means (4.78 ± 0.42, 4.69 ± 0.42, *p *= 0.043)

TSP-4 levels were significantly higher in Fontaine stage II (intermittent claudication, *n* = 181) vs. Fontaine stage I (asymptomatic patients) (4.78 ± 0.42, 4.69 ± 0.42, *p* = 0.043), as shown in Fig. 1. TSP-4 correlated significantly with ABI (*r* = − 0.141, *p* = 0.023, *n* = 259) after exclusion of patients with mediasclerosis. Patients with more advanced disease, as determined by an ABI cutoff of 0.5, displayed significantly higher TSP-4 values (ABI > 0.5, *n* = 235: 4.70 ± 0.43, ABI < 0.5: 4.96 ± 0.41, *p* = 0.005). No significant difference was seen in patients with (n = 92) or without mediasclerosis (4.78 ± 0.39, 4.72 ± 0.43, *p* = 0.262). Toe-brachial index (TBI) values were used to evaluate the association in mediasclerotic patients. No significant correlation could be seen in these patients (*r* = − 0.056, *p* = 0.613, *n* = 84). To further prove this observation, binary logistic regression on Fontaine I vs. II for TSP-4 levels was performed. In univariate fashion, the increase of log (TSP-4) by one unit showed an odds ratio (OR) of 1.67 (1.01–2.76) for the increase in the clinical stage. After adjustment for traditional risk factors (age, gender, LDL-C, hypertension, lipoprotein(a), diabetes, eGFR and smoking), the model remained significant with an OR of 1.70 (1.02–2.82; see Table 2). After adjustment for the same variables and exclusion of mediasclerotic patients in a linear regression model, each increase of one unit of (log) TSP-4 predicted a decrease in by 0.053 (Table 3).

**Table 2:** Multivariable binary logistic regression model for Fontaine I vs. II

	PAD I vs. II		
	B (SE)	OR (95% CI)	*p *value
log (TSP-4)	0.53 (0.26)	1.70 (1.02–2.82)	**0.043**
Age	− 0.01 (0.01)	0.99 (0.96–1.02)	0.407
Gender	0.29 (0.25)	1.34 (0.83–2.17)	0.233
LDL-C (mg/dl)	0.002 (0.003)	1.0 (0.99–1.01)	0.474
Lipoprotein(a) (mg/dl)	0.07 (0.10)	1.08 (0.86–1.30)	0.431
Hypertension	0.17 (0.43)	1.12 (0.52–2.74)	0.684
Diabetes status	0.11 (0.26)	1.11 (0.67–1.83)	0.680
eGFR	− 0.009 (0.007)	0.99 (0.98–1.01)	0.238
Smoking	0.21 (0.36)	1.23 (0.61–2.47)	0.565
Constant	− 2.25 (2.00)		0.261
Chi-Quadrat 0.456, *R*^2^ 0.036			

*p* < 0.05 significant (in bold)

**Table 3:** Multivariable (age, gender, LDL-C, lipoprotein(a), eGFR, hypertension, smoking status and diabetes status) linear regression model for ABI

	B	SE B	*p *value
Log (TSP-4)	− 0.053	0.026	**0.042**
*R*^2^ = 0.143			

Each increase of one unit of TSP-4 (logarithmically transformed) predicts a decrease in ABI by 0.053

*p* < 0.05 significant (in bold)

### TSP-4 and diabetes

**Fig. 2:**
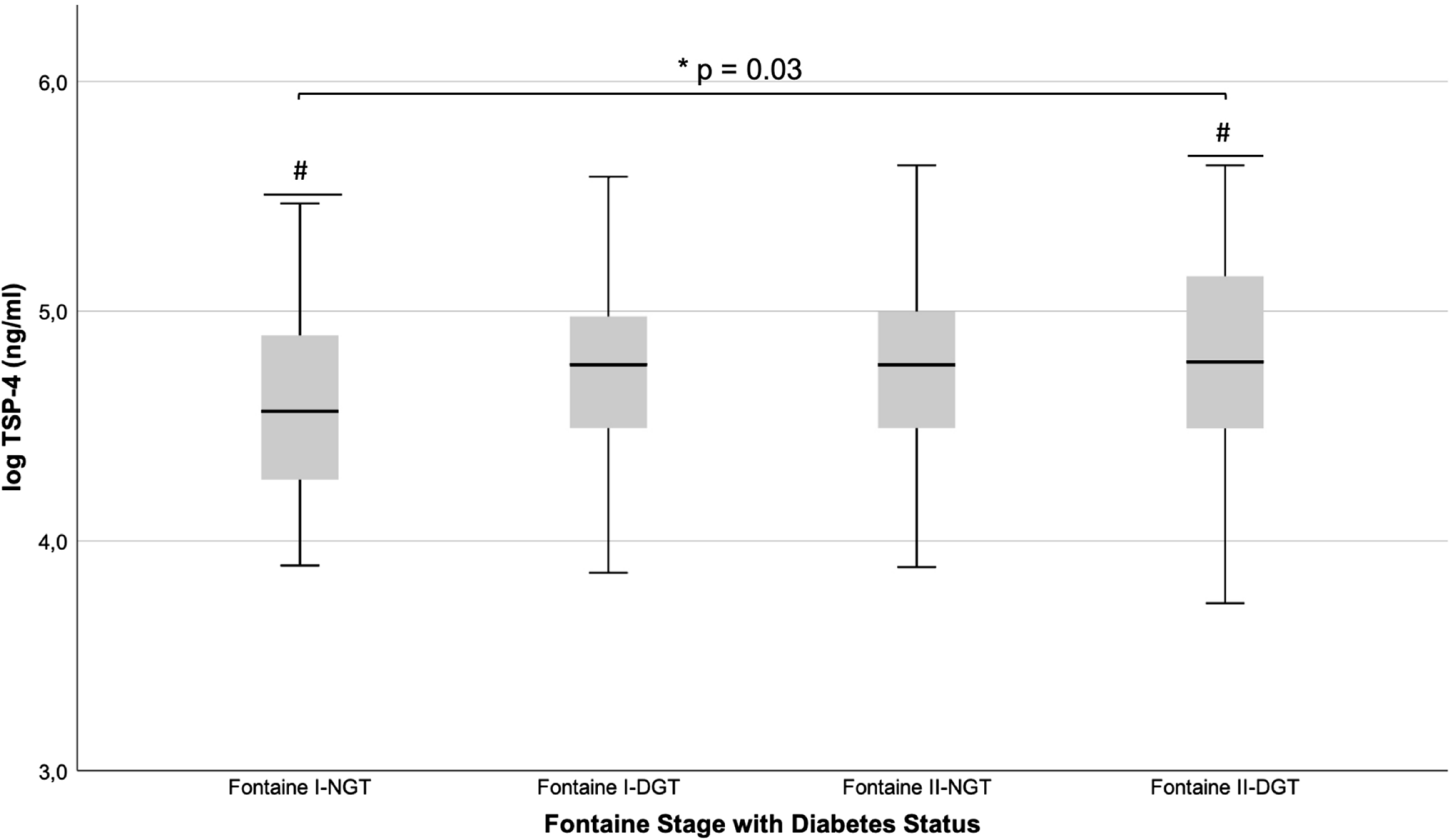
Boxplot for different PAD and glucose metabolism stages. *The overall model shows a significant difference in means (*p* = 0.03). ^#^Post hoc analysis shows a significant difference in means between PAD I-NGT (*n* = 55) vs. PAD II-DGT (*n* = 134) (4.59 ± 0.40, 4.79 ± 0.43, *p* = 0.015)

TSP-4 levels were higher in patients with DGT vs. NGT (4.76 ± 0.42 vs. 4.66 ± 0.41, *p* = 0.035). For further analysis of this observation, patients were split into 4 groups according to their Fontaine and diabetes status (Fontaine I-NGT *n* = 55, Fontaine I-DGT *n* = 129, Fontaine II-NGT *n* = 46, Fontaine II-DGT *n* = 134). One-way ANOVA showed a significant increase in TSP-4 levels between groups (*p* = 0.03) and mean values increased with a linear trend from patients with Fontaine I-NGT to Fontaine II-DGT. The highest difference in the post hoc analysis was found between Fontaine I-NGT vs. Fontaine II-DGT (4.59 ± 0.40, 4.79 ± 0.43, *p* = 0.015). Results of the post hoc analysis are depicted in Fig. 2.

## Discussion

This is the first study to demonstrate an association of TSP-4 with the severity of PAD. Levels of TSP-4 increased both with an impairment of the clinical stage classified by Fontaine and with decrease of ABI as a quantitative measurement. No significant association was observed between TSP-4 and TBI values of mediasclerotic patients. We assume that this is due to the rather small subgroup analysis with 84 available TBI values. In the subgroup analysis for PAD and status of diabetes, a linear increase in the TSP-4 levels was revealed from Fontaine I-NGT to Fontaine II-DGT. Associations with ABI and clinical stage of PAD withstood multivariable adjustment for traditional risk factors in regression analyses.

Clinical data on TSP-4 are limited. Our findings on higher levels in PAD as an occurrence of atherosclerosis are in line with the mouse models. In these models TSP-4 deficiency resulted in a decreased plaque burden [17]. Other members of the TSP-family have been linked to atherosclerosis in humans, such as TSP-1 [25] and TSP-2 [26]. Interestingly, TSP-4 has functions contrary to the other TSP family members in the vascular wall. While TSP-1 and TSP-2 mediate antiangiogenic effects [27, 28], TSP-4 induces angiogenesis [11, 13, 14]. The impact of angiogenesis on atherosclerosis has not been fully understood yet. The hypoxic trigger in a plaque reaching a specific size induces several inflammatory and angiogenic proteins, such as hypoxia-induced factor 1 (HIF1) and VEGF [29], as well as matrix-metalloproteinases (MMPs) and TSP-4. Newly built vessels in the plaque obtain a weak integrity and are prone to leakage [30]. Intraplaque haemorrhage causes migration of monocytes/macrophages as well as the deposition of haemoglobin, iron and cholesterol [31]. This process further leads to inflammatory upregulation by release of inflammatory cytokines and extracellular matrix proteins [32], eventually preceding to the full characteristics of a vulnerable plaque [5]. TSP-4 has been found to be abundantly present in atherosclerotic plaques [17] and our finding of a positive correlation between TSP-4 levels and the severity of PAD is in line with this pathophysiological concept. We hypothesize that the contrary functions of TSP-1/2 and TSP-4 should be seen separately. The antiangiogenic effects of TSP-1 and -2 might influence atherosclerosis by inhibition of vessel wall sprouting, while TSP-4-induced angiogenesis increases the plaque burden with the above-explained mechanisms. In the end, both effects might worsen the clinical stage at separate locations in the vessel wall.

Additionally, this study depicts a former unknown association of TSP-4 with diabetes. TSP-1 has previously been linked to type 2 diabetes mellitus. TSP-1 levels were increased in the vessel walls of diabetic Zucker rats [11]. On the molecular level, TSP-1 gene expression is induced through a hexosamine-dependent pathway. This leads to increased proliferation of smooth muscle cells of the human aorta (HASMC) [33] and might therefore account for the diabetic vascular complications. Molecular mechanisms for TSP-4 in diabetes are unknown. Since diabetes promotes microvascular angiogenesis in diabetic nephropathy [34] and retinopathy [35], we hypothesize that TSP-4 could be a marker of neovascular activity in atherosclerotic plaques of diabetic patients. The hyperglycaemic trigger given in diabetic conditions might therefore exaggerate plaque progression through enhanced TSP-4 expression. Further studies are needed to confirm this observation.

This study has several limitations. First, only patients with manifest peripheral arterial disease and stable disease were enrolled. Second, no causal role of TSP-4 in the atherosclerotic process could be demonstrated due to the study design. Thus, further studies to evaluate TSP-4 in other forms of atherosclerosis and critical limb ischemia are warranted.

In conclusion, our data demonstrates that TSP-4 is associated with more advanced PAD. This result is in accordance with previous experimental pre-clinical studies. Additionally, we found a relation between TSP-4 and diabetes. The combination of T2DM and intermittent claudication further increased TSP-4 levels. TSP-4 could be a novel marker for atherosclerotic burden, especially in the major subgroup of patients with concomitant diabetes. Further studies are needed to confirm these novel described associations.
